# A decontamination process adding a tensioactive agent and isopropanol to a closed-system drug transfer device for better control of isolator contamination. A prospective, parallel study

**DOI:** 10.1371/journal.pone.0201335

**Published:** 2018-08-08

**Authors:** Michèle Vasseur, Nicolas Simon, Chloé Picher, Camille Richeval, Marion Soichot, Luc Humbert, Christine Barthélémy, Sandrine Fleury-Souverain, Pascal Bonnabry, Bertrand Décaudin, Delphine Allorge, Pascal Odou

**Affiliations:** 1 Univ. Lille, EA 7365—GRITA—Groupe de Recherche sur les formes Injectables et les Technologies Associées, Lille, France; 2 CHU Lille, Institut de Pharmacie, Lille, France; 3 Univ. Lille, EA 4483 –IMPECS–IMPact de l’Environnement Chimique sur la Santé humaine, Lille, France; 4 CHU Lille, Pôle de Biologie-Pathologie-Génétique, Unité Fonctionnelle de Toxicologie, Lille, France; 5 Laboratoire de Toxicologie, Hôpital Lariboisière, Assistance Publique-Hôpitaux de Paris, Paris, France; 6 Pharmacy, Geneva University Hospitals and School of Pharmaceutical Sciences, University of Geneva, University of Lausanne, Geneva, Switzerland; Laurentian, CANADA

## Abstract

**Background:**

Despite the use of closed system drug transfer devices (CSTD), residual contamination from antineoplastic drugs is still detected inside isolators. The aim of this study was to compare the decontamination level obtained using a CSTD + standard cleaning procedure with a CSTD + standard cleaning procedure + specific decontamination procedure.

**Methods and findings:**

A comparative and prospective study was carried out in a newly opened compounding unit. Compounding was performed with a CSTD (BD-Phaseal, Becton-Dickinson). In the Control isolator (C), the cleaning process was completed daily with a standard biocide solution (Anioxyspray^TM^, Anios, France). In the Intervention isolator (I), weekly decontamination with a homemade admixture of sodium dodecyl sulfate 10^−2^ M/70% isopropanol (80/20, v/v) was added. Monitoring was performed via a validated LC-MS/MS method. Eight drugs (cyclophosphamide, cytarabine, dacarbazine, fluorouracile, gemcitabine, ifosfamide, irinotecan and methotrexate) were monitored daily over 14 consecutive weeks on three sites inside the isolators: gloves, workbench and window. Results are presented as the odds-ratio (OR) of contamination and as overall decontamination efficiency (Eff_Q_, %). The proportion of Eff_Q_ ≥ 90% was assessed by a Fisher’s exact test (p<0.05). Overall contamination rates (CR, %) were significantly different from one isolator to the other (CR_C_ = 25.3% *vs*. CR_I_ = 10.4%; OR = 0.341; p<0.0001). Overall Eff_Q_ values (median; 1^st^ and 3^rd^ quartiles) were higher in the intervention isolator (I: 78.3% [34.6%;92.6%] *vs*. C: 59.5% [-5.5%;72.6%]; p = 0.0015) as well as the proportion of days with an Eff_Q_ ≥ 90% (I: 42.9% *vs*. C: 7.1%; p = 0.077) but very variable depending on drugs.

**Conclusion:**

Adding a decontamination protocol with a tensioactive agent to a CSTD leads to better control of chemical contamination inside isolators. Improving decontamination by increasing decontamination frequency or modifying the protocol will be further studied.

## Introduction

The occupational exposure of healthcare workers to antineoplastic drugs was firstly described several decades ago [1]. Despite efforts to combat it, this topic remains an issue for healthcare workers [2]. The risks associated with occupational exposure to antineoplastic drugs can be genotoxic effects or a decrease in reproductive functioning [3,4]. Several sources of contamination have been determined in hospital settings: in pharmacies, notably on the external surface of commercial vials [5], during the compounding process [6], inside isolators [7] and in medical wards (e.g. preparations coming from the pharmacy and patients’ excreta) [8]. Soon after the first observation of this occupational exposure, professional recommendations were published [9]. Since then, they have been amended and improved. It is strongly advised today to wear personal protective equipment and use barrier isolators or laminar airflow hoods [10,11]. A regular update in healthcare workers’ training is also recommended [10,11].

In a compounding area, contamination may be translocated through direct contact with the contaminated surfaces of vials, by leaks or aerosolization or it may simply be spread by hands. Indeed, contamination has been detected on the ground in front of hoods or isolators and even on a computer mouse or phone [12].

It is therefore essential to decrease contamination resulting from the compounding process. In the past ten years, closed-system transfer devices (CSTD) have been promoted in guidelines [11]. These devices are intended to avoid direct contact between the preparation’s surroundings and antineoplastic drugs by “*mechanical prohibition of the transfer of environmental contaminants into the system and the escape of hazardous drug or vapor concentrations outside the system”* [13].

Recently, we have demonstrated that using a CSTD (BD-Phaseal, Becton-Dickinson, France) leads to a 50% reduction in the number of samples contaminated by ten cytotoxic drugs when compared to standard compounding devices (needles and mainly spikes) [6]. Although our results showed a significant difference in favor of the CSTD, residual contamination was still observed, as in other studies [14–16]. Furthermore, the efficacy of the CSTD is variable according to the drug tested. Means should therefore be reinforced to decrease contamination inside isolators and so reduce the probability of its spreading to surroundings.

Several studies have been carried out to find the best decontaminating solution [17–19]. It has previously been shown that decontaminating solutions including tensioactive agents could have a significant effect on residual contamination [19]. Tensioactive agents or surfactants are specific chemicals characterized by their structural bipolarity: one hydrophobic group and another hydrophilic group. They are classified as anionic, cationic or non-ionic surfactants and have various uses in healthcare, such as excipients to dissolve hydrophobic drugs in aqueous solutions or to compound injectable emulsions. Their interest in chemical decontamination is closely related to their ability to dissolve both hydrophilic and hydrophobic drugs, and then to be wiped on tissues. Queruau-Lamerie *et al*. established that solutions containing an admixture of anionic surfactant and isopropyl alcohol were effective in terms of surface decontamination [20]. These results paved the way for an assessment of a multifactorial approach combining the use of a CSTD with an optimized decontamination process.

The aim of the present study is then to assess the combination of a compounding process using a CSTD and a decontamination procedure including a solution specific to this same compounding process along with a standard cleaning process and to compare with the classic CSTD and standard cleaning procedure.

## Materials and methods

### Description of the compounding unit

The study was performed in a hospital compounding unit in which 45,000 preparations of cytotoxic drugs were compounded in 2016. The study started on December 16^th^ 2015 and was over on March 23^rd^ 2016. Three hospital pharmacists manage the compounding unit and 8.5 pharmacy technicians are employed in the compounding of antineoplastic drugs. All the pharmacy technicians are experienced personnel with CSTD knowledge and practice. This study took place just after the opening of the new compounding unit.

This unit is equipped with 4 new isolators (IM2111 and 2 IM1222, Sieve, Villeurbanne, France) and a total of 6 workplaces. Only the two IM1222 isolators (one workplace at each) were used in this study. One isolator was the "Control” (C) and the other the “Intervention” (I). The compounding process was performed on a BD-Phaseal (Becton Dickinson, Le Pont de Claix, France) in both isolators.

### Study design

#### Ethics statement

Lille University hospital benefited from a grant for this study. Since this study did not involve either human subjects or animals, no authorization had to be obtained from the ethics committee.

#### Distribution of compounded drugs inside isolators

Eight frequently prescribed drugs were chosen as contamination markers: cyclophosphamide, cytarabine, dacarbazine, 5-fluorouracile (5FU), gemcitabine, ifosfamide, irinotecan and methotrexate. To avoid any bias in their distribution inside the two isolators, it was decided to switch their compounding from one isolator to the other every day.

All preparations involving the monitored drugs were compounded with BD-Phaseal by specifically trained pharmacy technicians, who have been using this device in routine conditions for more than 1.5 years. So as not to interrupt the compounding activity essential for patients’ treatment, the other drugs were distributed among the four remaining workplaces.

The pharmacy technicians were assigned to one or the other workplace according to work organization in the unit.

#### Recording of critical incidents

To avoid bias in the study, any critical incident that could lead to chemical contamination was recorded. A critical incident was defined as vial breakage, spill or leak of drug inside either of the isolators.

#### Cleaning and decontamination procedures

The cleaning procedure inside isolators consisted of a daily cleaning process as well as a complete cleaning procedure followed by surface sterilization every 14 days. The daily cleaning procedure was performed at the same time each day in both isolators using a classic biocide (Anioxyspray^TM^, Anios, Lille, France), composed of ethanol (91.6 mg/g) and hydrogen peroxide (50 mg/g). This procedure was always performed after the morning compounding phase, corresponding to 80–90% of the daily production.

The fortnightly procedure involved a thorough cleaning of the whole isolator including the storage boxes inside the isolator with Anioxyspray^TM^ (Anios, Lille, France) followed by 90 mins’ exposure to vaporized H_2_O_2_. Neoprene gloves were changed on sterilization days unless a barrier disruption had occurred between two sterilization days.

Although the standard daily microbicide cleaning was the only cleaning procedure to remove both bacterial and chemical contamination in the two isolators, there was a difference from one isolator to the other in that a specific weekly decontamination process was added to the “intervention group” aimed at removing chemical contamination and adapted from Anastasi *et al*.’s research [21]. This protocol was implemented on Wednesdays, this being the day with the lowest compounding activity in the week. A solution mixture containing 80% of an aqueous solution of sodium dodecylsulfate (SDS) at 10^−2^ M and 20% of an aqueous solution of 70% isopropanol (IPA) was used [21]. After this decontamination process, the isolator was rinsed with water for injection to remove all potential residue and finally cleaned with classic biocide. The decontamination solution was prepared by the pharmacy each month under a laminar air-flow hood, conditioned in a sterile spray-bottle and tested to be bacteria-free before use, according to the European Pharmacopeia monograph 2.6.1 [22].

The decontamination procedures were performed by a pharmacy technician or by a pharmacist.

#### Contamination assessment

Both isolators were sampled on each working day over 14 consecutive weeks. The eight drugs were monitored in each isolator.

The assay used was the same as that described in a previous study which compared the contamination of isolators after using standard or BD-Phaseal devices [6]. Sampling was carried out daily with 100 μl water for injection and 5×5 cm compresses (ref. 22104KL1, Tetramedical, Annonay, France) inside the isolators. Three sites were defined at each workplace: worktop, inside face of window and gloves. Samples were taken on 10×10 cm surfaces (worktop and window) and on the whole gloves. This took place before and after the daily cleaning/decontamination procedure after preparing blank samples by filling a tube with a compress inside the isolator but without wiping. Samples were immediately frozen (-20°C) and analyzed within the following 14 days because of the instability of certain drugs (Nussbaumer *et al*., [23]).

Drugs were extracted from wipes with 2 mL of 0.1% formic acid in methanol over 20 minutes after placing 75 μL internal standard (clonazepam) solution on compresses. Samples were then centrifuged (4500 rpm for 10 min). After retrieving the wipes, solvent was evaporated at 40°C under nitrogen stream. The dry residual was dissolved with 100 μL 0.2% formic acid in acetonitrile.

The concentration was measured by liquid chromatography coupled to a tandem mass spectrometer (Xevo TQ-S, Waters, Guyancourt, France). Briefly, the analytes were separated over 4 minutes on a stationary phase Acquity UPLC C_18_ (1.8 μm, 2.1×150 mm) with an injection volume of 7.5 μL. The gradient mobile phase was composed of 5 mM ammonium formate buffer, 0.1% formic acid in water/0.1% formic acid in acetonitrile. The method was validated according to the technical guidelines provided by the French National Accreditation organism (COFRAC) for method validation in medical biology [24]. The lower limit of quantification (LOQ) was 1 ng (per analyzed surface) for cyclophosphamide, gemcitabine, ifosfamide and 10 ng for cytarabine, dacarbazine, fluorouracil, irinotecan and methotrexate. The limit of detection (LOD) was 1 ng for all drugs.

#### Outcomes and statistics

The primary outcome was the contamination rate (CR, %) inside isolators after decontamination. A sample was considered contaminated if at least one drug was detected after analysis. No contamination was recorded if the drug amount was less than 1 ng.

CR corresponds to the division of the number of samples with a detectable amount of drugs (> LOD) divided by the total number of measured samples. The study was designed to highlight a 50% difference in CR between the two isolators by using the following formula:

n=12×(zα2−z1−β)2(arcsinP1−arcsinP2)2 (1)

zα2 corresponds to type I risk and was fixed at 0.05, in a bilateral position;

z_1-β_ corresponds to type II risk and was fixed at 0.85;

P1 corresponds to the first proportion and was fixed at 12.2%, respecting the literature [6] and P2 was estimated at 6.1%. The target number of analyses to be computed was 780.

For any sample with contamination above the lower limit of quantification for the assay, contamination is expressed in ng. Contamination was assessed by the minimum, median, maximum and also the 1^st^ and 3^rd^ quartile values.

The secondary outcome was the decontamination efficacy of the studied solutions. Decontamination efficiency was computed for each drug (Eff_q_) or for all drugs (Eff_Q_) according to Anastasi *et al*., using the following formula:

$$Effq=1‑\frac{sum\;of\;contamination\;after\;cleaning\;(ng)}{sum\;of\;contamination\;before\;cleaning\;(ng)}$$ (2)

In the case of a higher contamination value after decontamination, negative values of Effq were replaced by 0%. As no toxicological reference values are currently available, our target for overall contamination was the lowest obtainable. In our institution, we therefore considered reasonable an Eff_Q_ value of at least 90%.

The study was performed under blind conditions. Results were communicated by the toxicology laboratory after the final sampling.

Statistics were established from the two isolators (Control *vs*. Intervention). Continuous data (e.g. handled doses) were compared by a Student’s t-test or a Mann-Whitney test. Categorical data (e.g. contamination rates) were compared by a *Chi*^2^ test or a Fisher exact test. All tests had a significance level of 5%.

## Results

### Study description

2,776 preparations containing the monitored drugs were compounded over 68 consecutive days. The number of preparations compounded was similar for the two isolators (1,417 in the Control isolator *vs*. 1,359 in the Intervention isolator) (Table 1). The delay (m±sd) between sampling and dosing days was compatible with drug stability [23]: 7.0±3.8 days with a maximum delay of 14 days for two sampling batches. A total of 1,088 samples were collected, including 272 blanks. The number of study samples was therefore 816. There was no significant difference in the mean compounded doses between the two isolators (Table 1). No critical incident occurred in either isolator during the study.

**Table 1:** Distribution of compounded doses (in mg) and number of preparations (N) in the two isolators. Results are given as mean±standard deviation.

	Control		Intervention		P
	Dose	N	Dose	N	
**5-fluorouracile**	2521±1728	504	2492±1730	446	0.796
**Cyclophosphamide**	1138±786	235	1151±793	209	0.868
**Cytarabine**	1524±1946	272	1470±2018	267	0.751
**Dacarbazine**	593.6±108.5	30	595.4±122.7	24	0.956
**Gemcitabine**	1712±329	159	1684±331	169	0.440
**Ifosfamide**	2842±2289	29	2105±1080	20	0.139
**Irinotecan**	295.4±61.4	124	286.59±64.05	169	0.235
**Methotrexate**	3015±2471	64	3358±2478	55	0.453

### Assessment of isolator contamination by antineoplastic drugs

#### Contamination analysis

Initial contamination was determined before the compounding unit opened and was confirmed to be null. Contamination levels for both isolators during the study are summarized in Table 2.

**Table 2:** Breakdown of contamination in control and intervention isolators. Data correspond to the minimum (min), 1^st^ quartile (Q1), median, 3^rd^ quartile (Q3) and maximum (max) values. Contamination values are expressed in ng.

	Control		Intervention	
	Before cleaning	After cleaning	Before cleaning	After cleaning
**Min**	3.7	3.6	0.0	0.0
**Q1**	82.3	39.8	42.4	3.1
**Median**	206.6	142.7	130.7	31.7
**Q3**	717.8	283.5	285.3	102.1
**Max**	11589	1848	2625	1627

During the study, contamination rates for blanks were 1.1% in the Control isolator and 0.8% in the Intervention isolator (p = 1.00). Overall contamination rates after the daily cleaning/decontamination process were significantly different for each group (CR_C_ = 25.3% vs. CR_I_ = 10.4%; Odds-Ratio = 0.341; p<0.0001).

As regards the surfaces tested, contamination rates were always significantly lower in the Intervention isolator after the cleaning process (Table 3). Contamination rates were different according to the surface tested: e.g. gloves were more contaminated than worktop or inside face of window.

**Table 3:** Contamination observed on gloves, worktop and window. Data are presented as overall contamination rates (CR, in %), Odds-Ratio (OR) and days without any contamination (N).

		Control		Intervention		OR before	OR after
		Before	After	Before	After		
**Gloves**	***CR***	36.0	27.7	22.9	13.8	0.530*	0.416*
	***N***	6	9	3	21	-	-
**Worktop**	***CR***	19.9	21.9	10.5	10.9	0.473*	0.434*
	***N***	16	13	29	27	-	-
**Window**	***CR***	30.3	26.3	11.8	6.4	0.306*	0.193*
	***N***	2	3	24	41	-	-

*p < 0.001 with a *Chi*^*2*^ test

**Fig 1:**
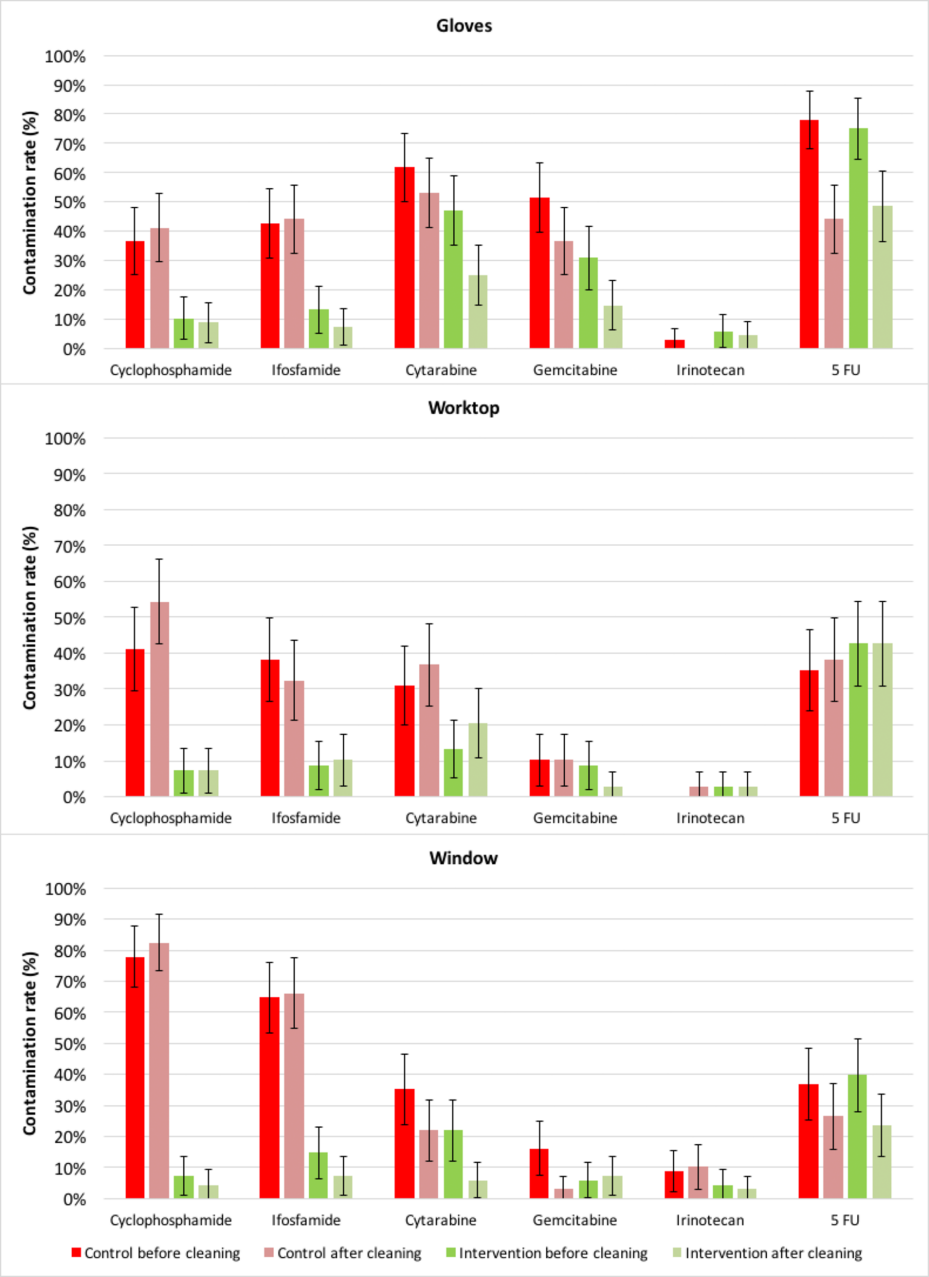
Contamination rates (in %) before and after the cleaning/decontamination process for the three studied surfaces.

Fig 1. indicates the CR for each drug in each group before and after the cleaning/decontamination process for each studied surface. Cyclophosphamide, ifosfamide, cytarabine, and gemcitabine had a lower CR after the cleaning process in the Intervention group on gloves. For the worktop, a decrease in CR is observed for gemcitabine after the decontamination process. On the window, the lowest CR is observed in the Intervention group after the decontamination process for all drugs except gemcitabine. The decontamination process had no particular effect on 5-FU contamination. Dacarbazine and methotrexate were never detected in the study.

Contamination amount generally appears to be lower after the decontamination process for each surface studied in the Intervention isolator compared to those in the Control isolator (Tables 4–6).

**Table 4:** Contamination values on decontamination days and decontamination efficiency (in %) on gloves. Contamination values (in ng) correspond to the sum of contamination measured before or after the cleaning/decontamination process.

	*Control*			*Intervention*		
*Day*	*Before*	*After*	*Eff*_*Q*_	*Before*	*After*	*Eff*_*Q*_
6	127.86	27.99	78%	2.76	0.00	100%
10	94.04	69.85	26%	51.29	0.00	100%
14	60.75	18.98	69%	180.43	0.00	100%
19	57.81	2.03	96%	99.09	41.21	58%
24	693.26	440.89	36%	0.00	0.00	-
29	201.13	14.61	93%	16.25	16.78	0%
34	37.51	1.43	96%	3.51	0.00	100%
38	416.70	172.56	59%	24.29	69.41	0%
43	68.25	560.80	0%	43.69	21.35	51%
48	275.32	304.04	0%	72.07	0.00	100%
53	0.00	0.00	-	149.27	0.00	100%
58	138.75	29.85	78%	150.89	26.75	82%
63	649.94	19.49	97%	107.40	0.00	100%
68	59.87	44.38	26%	334.61	386.94	0%

**Table 5:** Contamination values on decontamination days and decontamination efficiency (in %) on worktop. Contamination values (in ng) correspond to the sum of contamination measured before or after the cleaning/decontamination process.

	*Control*			*Intervention*		
*Day*	*Before*	*After*	*Eff*_*Q*_	*Before*	*After*	*Eff*_*Q*_
6	37.34	109.01	0%	121.40	20.42	83%
10	89.01	34.40	61%	0.00	14.55	0%
14	5.57	0.00	100%	236.06	0.00	100%
19	78.51	0.00	100%	0.00	0.00	-
24	179.49	22.84	87%	0.00	0.00	-
29	33.12	6.50	80%	0.00	0.00	-
34	10.46	0.00	100%	48.90	1.07	98%
38	81.52	34.90	57%	0.00	0.00	-
43	15.74	0.00	100%	0.00	0.00	-
48	59.30	9.27	84%	58.86	0.00	100%
53	5.97	0.00	100%	0.00	0.00	-
58	308.54	4.24	99%	0.00	0.00	-
63	0.00	23.86	-	0.00	0.00	-
68	12.11	1198.35	0%	0.00	76.86	-

**Table 6:** Contamination values on decontamination days and decontamination efficiency (in %) on window. Contamination values (in ng) correspond to the sum of contamination measured before or after the cleaning/decontamination process.

	*Control*			*Intervention*		
*Day*	*Before*	*After*	*Eff*_*Q*_	*Before*	*After*	*Eff*_*Q*_
6	37.34	31.66	15%	224.85	13.07	94%
10	89.01	57.78	35%	27.60	51.20	0%
14	5.57	1.10	80%	49.63	0.00	100%
19	78.51	72.88	7%	0.00	0.00	-
24	179.49	1383.88	0%	0.00	0.00	-
29	33.12	83.98	0%	12.65	0.00	100%
34	10.46	6.21	41%	12.99	0.00	100%
38	81.52	62.26	24%	0.00	0.00	-
43	15.74	5.95	62%	15.47	0.00	100%
48	59.30	83.02	0%	0.00	0.00	-
53	5.97	10.02	0%	0.00	0.00	-
58	308.54	17.74	94%	12.86	0.00	100%
63	0.00	0.00	-	43.73	0.00	100%
68	12.11	24.82	0%	0.00	28.67	-

#### Analysis of decontamination efficiency

A comparison of Eff_Q_ values for all study days (N = 68) with a non-parametric Mann and Whitney test results in a significant difference: the medians and [1^st^ and 3^rd^ quartiles] were 59.5% [-5.5%;72.6%] and 78.3% [34.6%;92.6%] for Control and Intervention groups, respectively (p = 0.0015). If there was no difference in the number of sampling days with Eff_Q_ ≤ 0% (Control: 19 *vs*. Intervention: 10; p = 0.096), a significant difference was observed for the number of sampling days with Eff_Q_ ≥ 90% (Control: 6 *vs*. Intervention: 21; p = 0.0005), or Eff_Q_ = 100% (Control: 0 *vs*. Intervention: 11; p = 0.0002). Decontamination efficiency (Eff_q_) was different depending on the drug analyzed. The values of Eff_q_ for 5FU, cytarabine, ifosfamide, cyclophosphamide and gemcitabine are reported in Table 7. In the case of irinotecan, more contamination was measured in the Control group after cleaning (mean: 64.0 ng; 6 days) than before (mean: 43.0 ng; 2 days) whereas in the Intervention group, contamination was lower after cleaning (26.6 ng; 2 days) than before (331.0 ng; 1 day).

**Table 7:** Overall decontamination efficiency (Eff_q_) between control and intervention groups. Eff_q_ (in %) is presented per drug as median [Q1; Q3]. P-values were obtained with a non-parametric Mann-Whitney test.

*Drug*	*5FU*	*Cytarabine*	*Ifosfamide*	*Cyclophosphamide*	*Gemcitabine*
***Control***	91% [69.1; 100]	71% [16.4; 97]	29% [2.5; 64]	6% [-47.3; 40]	88% [30.5; 100]
***Intervention***	100% [41.3; 100]	95% [63.2; 100]	90% [81.7; 96]	100% [58.0; 100]	100% [77.7; 100]
***P-value***	0.842	0.026	0.0007	0.0007	0.298

When focusing on decontamination days, the mean overall Eff_Q_ value was much higher in the Intervention isolator (I: 61.0±41.5% *vs*. C: 42.4±37.3%; p = 0.136), but very variable depending on drugs. Decontamination was more effective for both cyclophosphamide and gemcitabine. The proportion of days with an Eff_Q_ ≥ 90% was higher in the Intervention isolator (I: 42.9% *vs*. C: 7.1%; p = 0.077). Therefore, residual contamination remained (median: 19 ng).

Decontamination efficiency was also variable according to the tested surface (Tables 4–6). In the Control isolator, the highest decontaminating effect was observed for irinotecan on gloves and for gemcitabine on both worktop and window, whereas the lowest effect was observed for gemcitabine on gloves, for cyclophosphamide on worktop and for cytarabine on window. In the Intervention isolator, the highest decontaminating effect was noted for ifosfamide on gloves and for irinotecan on both worktop and window, whereas the lowest was to be found for cyclophosphamide on gloves, for 5FU on worktop and for gemcitabine on window.

## Discussion

Various means have to be combined to ensure maximum control of chemical contamination by antineoplastic drugs inside isolators. Three main sources of contamination have been identified: the outside surface of vials, the compounding process (leakage and aerosolization) and spreading by contaminated hands. The results of the present study show a significant difference between the two strategies tested. Indeed, the combination of a CSTD + daily cleaning + weekly decontamination process led to a significant reduction in isolator contamination compared to the association of a CSTD + daily cleaning only, especially for cyclophosphamide, ifosfamide, cytarabine and gemcitabine.

Contamination evolution was very variable throughout the study in both isolators, but showed a decreasing trend over time. Contamination measured after cleaning/decontamination was generally lower than contamination measured before, although sometimes it could be higher. This phenomenon has already been reported elsewhere [21]. If there is no logical explanation, it may be hypothesized that the cleaning process is sometimes involved in spreading contamination rather than desorbing drugs. Nevertheless, contamination was shown to be globally lower in the Intervention isolator, both for the decontamination days and for the periods between two decontamination days. The use of a specific cleaning solution helped to reduce the occurrence of inefficiency (Eff_Q_ ≤ 0%) and significantly increased the number of days with high Eff_Q_ (Eff_Q_ ≥ 90%).

Overall CR before cleaning was observed to be much lower in the Intervention group. As CR reflects the cumulative effect of residual contamination after cleaning/decontamination and contamination generated during compounding, it is likely that the CR observed in the Intervention isolator is a consequence of the decontamination process and the compounding. As the compounding underwent no modifications (same drug vials, same pharmacy technicians…), it can be asserted that the CR was due to decontamination.

The study was performed inside isolators regularly sterilized by vaporized H_2_O_2_. If regular sterilization is always programmed, impromptu sterilization may be required in the case of sterility disruption. No specific effect on contamination was expected, as already indicated in a previous publication [25].

When focusing on the contamination rates observed on decontamination days, contamination was significantly lower in the Intervention isolator over the whole study period. However, the difference was not always significant, showing how dynamic contamination evolution is in relation to time, as demonstrated earlier by Anastasi *et al*. [21].

The difference observed was also dependent on the tested drug and surface. Even if slight differences were observed in decontamination efficacy between this study and that of Queruau-Lamerie *et al*., decontamination efficacy levels are close to those previously obtained during simulated contamination studies [20], except for cytarabine whose decontamination efficacy was significantly lower in this study. This difference could be due to several factors, such as the difference in contamination amount, surface type or wipes used.

The analytical assay used for contamination measurement was the same as the one used in a previous study [6]. Although blank samples were not contaminated before the study began, a few blank samples became contaminated during the study, probably due to handling errors during sampling. As shown in our results, the contamination rates of blank samples were very low and no difference was evident between the two isolators.

There was therefore a reduction in the number of contaminated samples in the Intervention isolator, leading to the conclusion that the addition of a weekly decontamination process may provide better control of residual contamination inside isolators than simply the use of a CSTD and daily cleaning with a microbicide solution. This study also shows that standard biocide solutions are not effective in removing chemical contamination. In our previous study which aimed at assessing the effect of implementing a CSTD in the compounding process when standard biocide is used as decontaminating agent, the sum of contamination observed after cleaning ranged between 17–4400 ng and 0–2250 ng in the groups using standard devices and CSTD, respectively [6]. In the present study, the sum of contamination observed after decontamination/cleaning ranged from 8 to 1850 ng in the Control isolator and 0 to 490 ng in the Intervention isolator. This confirms previously published data.

Efficacy in decontaminating surfaces depends on the decontaminating solution used, and also on the surface to be wiped [17–19,26]. The choice of solution containing SDS and IPA was related to previously published data [17,20] as it proved to be effective on several drugs and several surfaces. In this study, the CR% observed after the cleaning process was reduced by 59% in the Intervention isolator. The OR was 0.341, resulting in a 61.5% reduction in contamination risk. The effect differed depending on the surface: the association of a CSTD + cleaning process + decontamination was better on window > gloves > worktop. This could probably be explained by the fact that wiping is easier on flat surfaces, less exposed to direct contamination (e.g. window) than others, and by the polymer on the surface. On the other hand, the effect was positive on gloves, so decreasing the risk of skin exposure for pharmacy technicians. Moreover, neoprene gloves were changed regularly (every 14 days if no incident) before isolator sterilization.

Considering a target contamination of 10 ng as suggested by the MEWIP project [27], a contamination sum lower than 10 ng after the cleaning process was more frequently observed in the Intervention isolator (data not shown), but it was nevertheless over 10 ng on many study days. These results are consistent with previously published data. The effect of decontamination is variable, which is why, depending on the origin of the contamination (drug type) and on the nature of the surface to be cleaned, the Eff_Q_ did not reach 100% on each decontamination day.

In this study, 5FU was identified as being the most contaminating drug in the Intervention isolator after poor decontamination/cleaning efficacy of the SDS solution. Its contamination percentage was 55.8%. Such a result was clearly unexpected considering previously published data [17,18]. In their simulated contamination study, Böhlandt *et al*. tested three solutions (i.e. distilled water, 10^−2^ M SDS/70 IPA -80/20- and towelettes soaked with propan-1-ol and propan-2-ol) on four surfaces (glass, stainless steel, polyvinyl chloride and laminated wood), different from ours, with 1 ng/cm^2^ of 5-FU [17]. An Eff_q_ of over 99% was observed on a glass surface with the 3 solutions. Eff_q_ levels of 95–99% were observed on PVC and stainless steel surfaces with the three tested solutions. Whatever the decontaminant used on laminated wood, Eff_q_ was under 95%. In their study, Cox et *al*. tested a marketed towelette kit soaked with a quaternary ammonium solution and isopropanol on a stainless steel surface to obtain an Eff_q_ of over 98% [18].

Decontamination efficiency depends on the drug (type and amount), the decontaminating solution, the surface type, but also on the analytical technique used to quantify drugs. In the case of 5-FU, which is a very hydrophilic drug, dosing remains difficult with a reverse-phase LC-MS method, because of its low quantitative performances (in terms of precision, recovery and sensitivity). Most samples had detectable, but unquantifiable, 5-FU amounts (results between LOD and LOQ) in both groups, so that no relevant conclusion could be reached. The results observed on decontamination efficiency on decontamination days require specific study to be clearly understood.

## Conclusion

This study shows that the use of a specific decontamination solution, including a surfactant and an alcoholic solvent, results in decreasing chemical contamination inside isolators, although it cannot eliminate it totally. Further studies are required to gain better control of all sources of contamination, by reinforcing decontamination methods inside isolators.
